# Crocin’s effect on phenotype switching of J774A.1 macrophages depends on their polarization state during exposure

**DOI:** 10.22038/IJBMS.2023.70859.15392

**Published:** 2023

**Authors:** Hakimeh Abdi, Marjan Roshanravan, Farshad Mirzavi, Hossein Hosseinzadeh, Fatemeh Mosaffa

**Affiliations:** 1 Department of Pharmaceutical Biotechnology, School of Pharmacy, Mashhad University of Medical Sciences, Mashhad, Iran; 2 Cardiovascular Diseases Research Center, Birjand University of Medical Sciences, Birjand, Iran; 3 Pharmaceutical Research Center, Pharmaceutical Technology Institute, Mashhad University of Medical Sciences, Mashhad, Iran; 4 Department of Pharmacodynamics and Toxicology, School of Pharmacy, Mashhad University of Medical Sciences, Mashhad, Iran; 5 Biotechnology Research Center, Pharmaceutical Technology Institute, Mashhad University of Medical Sciences, Mashhad, Iran

**Keywords:** Arg1, Crocin, Inflammation, Macrophage, Nos2, Saffron

## Abstract

**Objective(s):**

Macrophages exhibit versatile phenotypes, with M1 macrophages releasing inflammatory cytokines and possessing microbicidal activities, while M2 macrophages release anti-inflammatory cytokines and contribute to tissue repair. The M1/M2 imbalance plays a significant role in various pathological processes. Crocin, known for its antioxidant properties and ability to eliminate free radicals, has been investigated for its potential anti-inflammatory effects. We examined the effect of the primary activation state of macrophages on their phenotype switching when exposed to crocin.

**Materials and Methods:**

The crocin impact on macrophage viability was evaluated by MTT. TNF-α, IL-6, and IL-10 secretion, as well as *Nos2/Arg1* ratio, were measured in cells treated with crocin or LPS+IFN-γ (M1 inducers), in cells concurrently treated with crocin and LPS+IFN-γ or in cells pretreated with crocin before M1 induction.

**Results:**

Crocin did not show any toxicity at the concentration of 500 µM or lower. When uncommitted macrophages were exposed to crocin (25-100 µM), it elevated certain M1 activity indicators, including *Nos2/Arg1* ratio and TNF-α secretion, but not IL-6. Crocin in concurrent treatment with LPS+IFN-γ prevented the increase in M1 indicators, *Nos2/Arg1* ratio, and TNF-α secretion. However, pretreatment of cells with crocin before the addition of LPS+IFN-γ did not reverse M1 induction in macrophages; instead, it further increased the *Nos2/Arg1* ratio and TNF-α secretion. IL-10 was not detectable in any of the experimental groups.

**Conclusion:**

It appears that the modulatory effects of crocin on macrophage M1/M2 phenotype switching partly depend on the presence or absence of inflammatory mediators and, accordingly, the initial state of macrophage commitment.

## Introduction

Macrophages are differentiated immune cells that exist widely in the body and are primarily responsible for maintaining homeostasis and combating pathogen invasion (1). They function as immune control switches, balancing pro-inflammatory and anti-inflammatory activities. Primary macrophages found in different tissues undergo polarization based on changes in their environment, resulting in the formation of different subtypes, namely M1 and M2 macrophages (2). M1/M2 ratio represents the opposing activities of macrophages (3). Polarized M1 cells are strong defenders against microbes and produce pro-inflammatory cytokines, such as TNF-α and IL-6. When macrophages are in the polarized M1 state, their focus shifts from promoting cell growth and division to executing immune defense functions. On the other hand, polarized M2 cells secrete anti-inflammatory cytokines such as IL-10, and their activity promotes tissue repair and cell proliferation (4, 5). Recent studies have examined metabolic changes in different macrophage phenotypes including the metabolism of arginine, glutamine, serine, and glycine amino acids (6). Depending on the stimulus, arginine can be metabolized in two different enzymatic pathways: Nitric oxide synthase-2 (*Nos2*) and arginase-1 (*Arg1*), which are characteristic markers of the M1 and M2 phenotypes of macrophages, respectively (7). Thus, the M1/M2 classification of macrophages can be summarized by these two differing arginine metabolic processes (8). It has been observed that during the macrophage polarization process, there is a change in the ratio of the *Nos2*/*Arg1* markers (9-12). M2-activated macrophages increase the expression of *Arg1*, resulting in a shift in their Nos2/Arg1 ratio and reduced nitric oxide (NO) production. Conversely, M1 macrophage activation leads to an increase in this ratio (13). 

Changes in the balance between M1 and M2 macrophage responses play a crucial role in various disorders including infections, cancers, autoimmune diseases, and atherosclerosis (14). For instance, both M1 and M2 macrophages are present in atherosclerotic plugs, and a progressive switch from M2 to M1 has been observed during Atherosclerotic plaque formation (15). Therefore, modifying the direction of macrophage polarization toward the desired phenotype in diseased tissues could potentially improve the condition, leading to a healthier state of the tissue. Carotenoids, the bioactive compounds found in many herbal foods, have been found to modulate inflammation and immune processes (16). Among the approximately 700 carotenoids synthesized by photosynthetic algae, plants, and bacteria, around 50 are consumed by humans, and some can be detected in human blood and tissue (17). Saffron, the dried stigma of the *Crocus sativus* plant, has been widely used as an herbal dye and spice for many years and is recognized in some countries as traditional medicine due to its effectiveness in alleviating various diseases. One of the carotenoids present in saffron is crocin (mono and diglycosyl esters of polyene dicarboxylic acid known as crocetin) which possesses health benefits, including anti-inflammatory, antioxidant, memory-enhancing, anti-tumor, and antidepressant effects. Crocin has demonstrated high efficacy and low toxicity in laboratory animal models (18). The potential for free radical scavenging and the antioxidant properties of crocin has been suggested to be implicated in its anti-inflammatory attributes (19). 

Existing evidence suggests that macrophage polarization is a complex process influenced by numerous factors, leading to diverse activation outcomes. Even after acquiring a specific phenotype, macrophages possess the capability to adapt and undergo further changes in response to new environmental stimuli. This indicates that polarization is reversible, allowing macrophages to switch between different functional states (20). Therefore, it is crucial to elucidate the conditions that influence the effects of crocin on M1 and M2 balance in order to design therapeutic strategies that utilize the immunomodulatory properties of crocin. In this study, we investigated, for the first time, the influence of the primary commitment state of macrophages (uncommitted or M1 commitment states induced by LPS and IFN-γ) at the time of exposure to crocin on the outcome of phenotype switching in J774A.1 murine macrophages. We assessed the secretion of IL-10, IL-6, and TNF-α, as well as the *Nos2*/*Arg1 *ratio which are well-known indicators of macrophage polarization status.

## Materials and Methods

***Cell culture and treatment***

J774A.1 murine macrophage cell line was purchased from the Pasteur Institute of Iran (Tehran, Iran). The cells were cultured in DMEM (Dulbecco’s Modified Eagle Medium) high glucose supplemented with 10% (v/v) FBS, 100,000 U/L penicillin, and 100 mg/L streptomycin, at 37 ^°^C in a humidified incubator (5% CO_2_, 95% air). 

***Cell viability assay***

Macrophage cells were seeded at a density of 5.0×10^3^ in per well in 96-well microplates for 48 hr. After 24 hr treatment with different concentrations of crocin (10, 20, 50, 100, 250, and 500 μM), the medium was removed and the wells were washed twice with PBS. MTT assay was performed. MTT solution (3-(4, 5-dimethylthiazol-2-yl)-2,5-diphenyl tetrazolium bromide)(5 mg/ml of culture medium) was added to each well, and the plates were maintained for 4 hr at 37 ^°^C and 5% CO_2_. Formazan crystals were dissolved using DMSO (Dimethyl sulfoxide), and the optical density (OD) was measured at 570 nm. 

***Cytokine secretion measurements***

Macrophages were cultured at a density of 4.0×10^5^ cells/ml per well of 6-well plates for 48 hr. Crocin was dissolved in a fresh cell culture medium daily. Experimental groups were: the cells exposed to only culture medium (untreated cells), the cells treated for 12 hr with LPS (100 ng/ml)+IFN-γ (20 ng/ml) (for M1 activation), the cells treated with crocin (25, 50, and 100 μM) for 24 hr the cells simultaneously treated with crocin (25, 50 or 100 μM) and LPS+IFN-γ for 24 hr (concurrent treatment), the cells treated with crocin (25, 50 or 100 μM) for 24 hr and then LPS+IFN-γ for additional 12 hr (pretreatment with crocin).

The levels of cell-released TNF-α, IL-6, and IL-10 were measured in the collected supernatants of untreated and treated cultured cells, using enzyme-linked immunosorbent assay (ELISA) (Invitrogen, USA), following the manufacturer’s instructions. Optical density was evaluated by the Epoch™ Microplate Spectrophotometer (BIO TEK, Instruments, USA).

***Arg1 and Nos2 mRNA expression analysis***

Macrophages were cultured at a density of 2.0×10^5^ cells/ml per well of 12-well plates for 48 hr. The cells were treated according to the protocol described in the cytokine secretion measurement section, and crocin was applied at a concentration of 50 μM in crocin-containing treatments. FavorPrep Blood/ Cultured Cell Total RNA Mini Kit (Favorgen, Taiwan) was used for total RNA extraction, according to the manufacturer’s protocol. Total RNA (2 µg) and cDNA Synthesis Kit (YTA, Iran) were used to synthesize cDNA with the following temperature program: 70 ^°^C for 5 min, 42 ^°^C for 60 min, and the reaction was terminated by heating at 75 ^°^C for 5 min. QPCR (quantitative polymerase chain reaction) was performed using SYBR Green qPCR Master Mix 2X (YTA, Iran) in a Real-Time PCR machine (Step One Applied Biosystem, USA). The reaction mixture in each qPCR tube with a total volume of 20 μl, included: 2 μl of cDNA, 10 μl of SYBR Green mixture, 0.4 μl of each primer (forward and reverse primer of genes of interest)(10 μM), 0.4 μl of a passive reference dye, and 6.8 μl of nuclease-free water. qPCR was performed under the following conditions: initial denaturation (95 ^°^C for 3 min) and amplification for 40 cycles (95 ^°^C for 5 sec for denaturation, 60 ^°^C for 60 sec for annealing and extension) followed by 95 ^°^C for 15 sec, 60 ^°^C for 60 sec and 95 ^°^C for 15 sec for melting curve setting. Each Ct (threshold cycle) value of the target genes was normalized to β-Actin Cts as a reference gene. The efficiency values of reference and target gene amplification reactions were between 90 and 110%. Consequently, fold changes of *Arg1* and *Nos2* mRNA were calculated using the 2^-ΔΔct^ method. The primer sequences used in this study are provided in Table 1. 

***Statistical analysis***

All data were reported as mean±standard error of the mean (SEM) of three independent experiments (n=3). Statistical analysis was performed using one-way ANOVA followed by Tukey-Kramer *post-hoc* test and Dunnett’s multiple comparisons test for MTT assay, using the Prism 7 software package. Brown-Forsythe and Shapiro-Wilk tests were used to assess variance equality and verify the normality of the data, respectively. A *P*-value<0.05 was considered to be statistically significant.

## Results

***Effect of crocin on macrophage viability***

**Figure 1:**
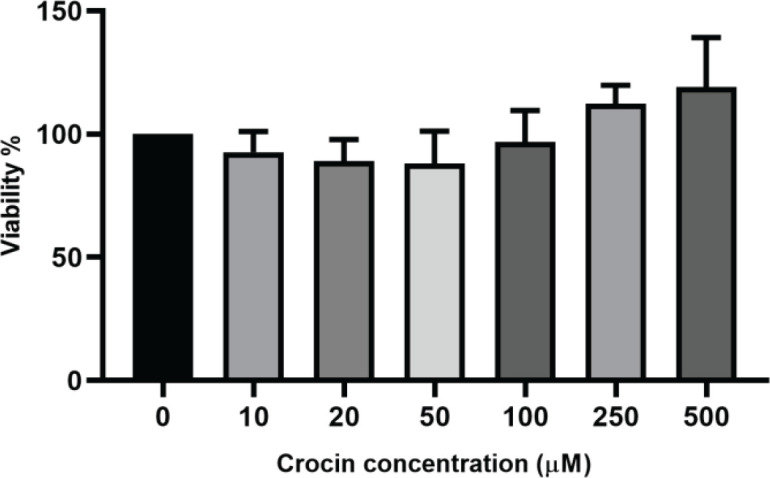
Effects of crocin on macrophage viability

The results of the MTT assay showed that treatment of macrophage cells with crocin for 24 hr at concentrations ranging from 10 to 500 μM had no toxic effect on macrophages (Figure 1, *P*=0.0135).

***Proinflammatory cytokine secretion analysis***

*LPS+IFN-γ treatment*

**Figure 2:**
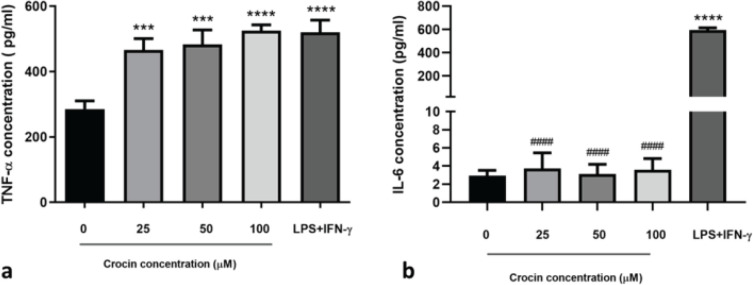
Effects of crocin on IL-6 and TNF-α secretion

Treatment of cells with LPS (100 ng/ml)+IFN-γ (20 ng/ml) for 12 hr significantly increased the secretion of TNF-α and IL-6 proteins (about 1.8- and 202-mean fold changes, respectively) in comparison to untreated cells (Figure 2a and 2b, *P*<0.0001). IL-10 was undetectable in the supernatant of untreated and LPS+INF-γ-treated cells.

*Crocin treatment*

TNF-α secretion by macrophages treated with different concentrations of crocin (25-100 μM) for 24 hr was significantly higher (1.63 to 1.84- fold) than that of untreated cells. Crocin exhibited comparable potency to LPS+IFN-γ in inducing TNF-α secretion (Figure 2a, *P*<0.0001). 

Crocin did not cause any changes in the secretion of IL-6 compared to untreated cells (Figure 2b, *P*<0.0001).

IL-10 was undetectable in the supernatant of the crocin-treated cells.

*Crocin pretreatment*

**Figure 3:**
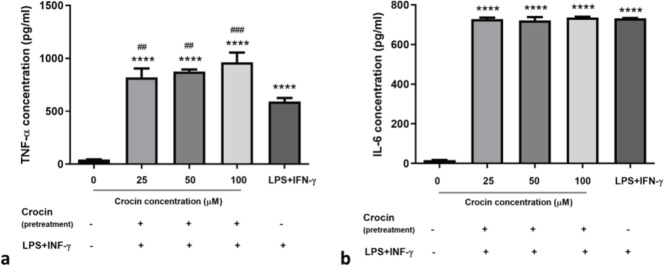
Effects of crocin pretreatment on IL-6 and TNF-α secretion

Pretreatment of macrophages with crocin (25-100 µM) for 24 hr before exposure to LPS+IFN-γ, significantly increased TNF-α secretion compared to untreated cells (about 19- to 22-mean fold change,* P*<0.0001) and even compared to M1 activated cells (1.4-1.6-fold,* P*<0.005) (Figure 3a).

Crocin-pretreated cells (25-100 µM) exhibited higher levels of IL-6 secretion (about 43-fold) compared to untreated cells (*P*<0.0001). Crocin exposure before LPS+IFN-γ treatment could not alleviate the increased expression of IL-6 induced by M1 activation (Figure 3b). IL-10 was not detected in the supernatant of crocin-pretreated cells.

***Concurrent treatment with crocin and LPS+IFN-γ***

**Figure 4:**
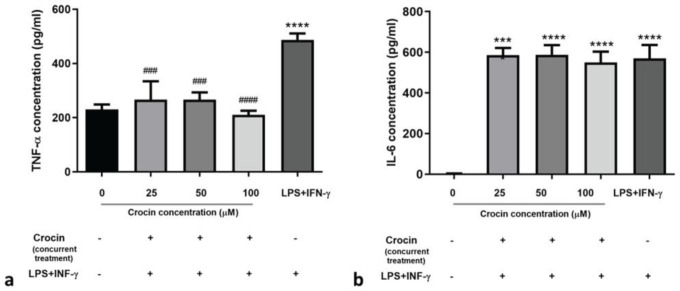
Effects of concurrent treatment of crocin and LPS+IFN-γ on IL-6 and TNF-α secretion

In cells treated with crocin and LPS+IFN-γ simultaneously, TNF-α secretion was significantly lower (about 0.4 to 0.5-fold) than that of M1-activated cells (*P*≤0.0001)(Figure 4a). The presence of crocin in concurrent treatment could not prevent the escalation of IL-6 in the supernatant of the cells induced by LPS+IFN-γ. Co-treated cells showed higher levels of IL-6 in their supernatants (163- to 165-fold) compared to the untreated cells (*P*<0.0001)(Figure 4b).

***Effect of crocin on Arg1 and Nos2 mRNA expression and their ratio***

*Nos2*

**Figure 5:**
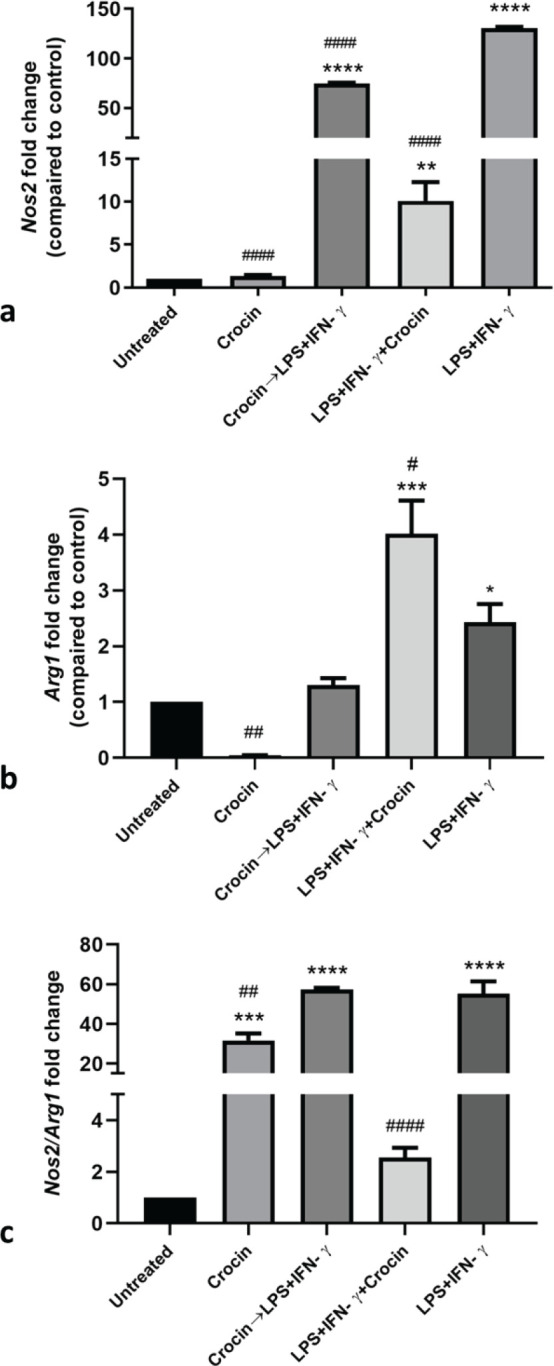
Evaluation of *Arg1* and *Nos2 *mRNA expression levels

M1 activated macrophage cells exhibited a significant increase (130.3±0.3-fold change) in *Nos2* mRNA expression level (*P*<0.0001). Crocin concurrent treatment with M1 inducers could diminish this *Nos2*-expression enhancement to about one-tenth (10.1±0.4-fold change, *P*<0.0001). In cells pretreated with crocin before exposure to LPS+IFN-γ, *Nos2* expression increased remarkably compared to untreated cells (73.8±0.06-fold change, *P*=0.0001), although this increase was significantly lower than M1-activated cells (about 0.6 fold lower, *P*<0.0001). No substantial change in *Nos2* was detected in crocin-treated cells (Figure 5a). 

*Arg1*

Treatment of macrophages with LPS+IFN-γ increased *Arg1* expression compared to untreated cells (2.433±0.06-fold change, *P*=0.0489). Cells treated with crocin showed a significant reduction in *Arg1 *mRNA expression level compared to untreated cells (about 0.04-fold, *P*<0.0019). *Arg1 *relative expression was also increased in the cells treated with crocin and LPS+IFN-γ simultaneously compared to both untreated cells (4.0±0.1-fold change*, P*=0.0003) and LPS+IFN-γ treated ones (about 1.65-fold change, *P*=0.0280). In crocin-pretreated cells, no significant change of *Arg1 *was observed compared to both untreated cells and M1-activated cells (Figure 5b). 

*Nos2/Arg1*

In comparison to the uncommitted cells (ratio=1), M1 activation by LPS+IFN-γ significantly increased the ratio to 55.2±1.2, *P*<0.0001. Pretreatment of the cells with crocin could not reverse this increase (57.5±0.1, *P*<0.0001). Crocin (50 μM) itself elevated the ratio to 31.7±0.7, *P*=0.0004. The concurrent treatment restored the raised ratio by LPS and IFN-γ to the levels close to untreated cells (2.5±0.07, *P*=0.0001) (Figure 5c). 

**Table 1:** List of primers sequences used for qPCR experiments

**β-Actin**	5'- GGC TGT ATT CCC CTC CAT CG -3'	5'- CCA GTT GGT AAC AAT GCC ATG T -3	(21)
***Arg1***	5'- CTC CAA GCC AAA GTC CTT AGA G -3'	5'- AGG AGC TGT CAT TAG GGA CAT C -3'	(22)
***Nos2***	5'- ACA TCG ACC CGT CCA CAG TAT -3'	5'- CAG AGG GGT AGG CTT GTC TC -3'	(23)

After exposure to crocin (10-500 µM) for 24 hr, cell viability was evaluated using an MTT assay. All data were reported as mean±SEM of four independent experiments (n= 4)

Following 24 hr treatment with crocin (25-100 μM), cytokine concentrations in the supernatant were measured using ELISA methods. (a) TNF-α; (b) IL-6. All data were reported as mean±SEM of 3 independent experiments (n= 3). (****, ####) *P<*0.0001, (***) *P=*0.0004 for crocin 25 μM and *P=*0.0002 for crocin 50 μM. The significant difference with untreated group (*) and LPS+ IFN-γ (#)

Following 24 hr treatment with crocin (25-100 μM), macrophages were treated with IFN-γ (20 ng/ml) and LPS (100 ng/ml) for an additional 12 hr. Cytokine concentrations in the supernatant were measured using ELISA methods. (a) TNF-α; (b) IL-6. All data were reported as mean±SEM of 3 independent experiments (n= 3). (****) *P<*0.0001, (###) *P=*0.0001, (##) *P=*0.0051 for crocin 25 μM and *P=*0.0010 for crocin 50 μM. The significant difference with the untreated group (*) and LPS+ IFN-γ (#)

Following 24 hr treatment with crocin (25-100 μM), and IFN-γ (20 ng/ml)+LPS (100 ng/ml), cytokines concentrations in the supernatant cells were measured using ELISA methods. (a) TNF-α; (b) IL-6. All data were reported as mean±SEM of 3 independent experiments (n=3). (####, ****) *P<*0.0001, (###,***) *P=*0.0001. The significant difference with the untreated group (*) and LPS+ IFN-γ (#)

Following treatment, pretreatment, and concurrent treatment with crocin 50 μM for 24 hr and IFN-γ (20 ng/ml) and LPS (100 ng/ml) for 12 hr *Arg1* and *Nos2* mRNA expression were measured using the RT-qPCR method. (a) *Nos2* relative mRNA expression; (b) Arg1 relative mRNA expression; (c) Nos2/Arg1 expression ratio. All data were presented as mean±SEM of three independent experiments (n=3). (####, ****) *P<*0.0001, (***) *P=*0.0003 in a and *P=*0.0004 in b, (**) *P=*0.0032, (##) *P=*0.0019 in b and *P=*0.0030 in c , (*) *P=*0.0489, (#) *P=*0.0280. The significant difference with the untreated group (*), and LPS+IFN-γ (#)

## Discussion

For a long time crocin has been known for its anti-inflammatory effects (24). In this study, for the first time, we examined whether the primary commitment status of macrophages plays a role in the ultimate impact of crocin on their phenotype switching. 

We used a combination of LPS and IFN-γ to simulate the inflammatory conditions and induce the M1 phenotype in macrophages (25). This activation significantly increased IL-6 and TNF-α secretion and the *Nos2*/*Arg1* ratio. Simultaneous treatment of macrophages with crocin and LPS/IFN-γ did not result in any significant change in TNF-α secretion and the *Nos2*/*Arg1* ratio. It has been previously described that downstream of LPS signaling pathway reactive oxygen species (ROS) are produced, which mediate the production of pro-inflammatory cytokines leading to the induction of M1 phenotype rather than M2 (26). Therefore, it is expected that antioxidant substances such as crocin can modify macrophage responses to LPS probably by scavenging the produced ROS and discontinuing the transduction of the signal to the downstream mediators of the inflammatory response. The ability of crocin to reduce TNF-α secretion and the *Nos2/Arg1 *ratio in macrophages in the presence of LPS+IFN-γ may partly explain why crocin shows therapeutic benefits in inflammatory diseases through the modification of the overriding M1 inflammatory state of macrophages in these diseases (27). In cultured rat brain microglial cells, crocin and crocetin (another ingredient of saffron and an important active metabolite of crocin in the body) were effective in inhibiting LPS-induced NO release. These compounds reduced the production of TNF-α, IL-β, and ROS following LPS stimulation in the cells (19) 

The anti-inflammatory potential of crocin has also been reported in inflammatory neurological diseases such as multiple sclerosis (28). Crocin has also been shown to reduce the enhanced ratio of Th1/Th2 induced by Concanavalin A (29). 

In concurrent treatment with crocin and LPS+IFN-γ, crocin could not modify IL-6 secretion in the J774A.1 macrophages. LPS and IFN-γ bind to Toll-like receptors 4 (TLR4) and IFN-γ receptors on the macrophage, respectively (26). It has been reported that following the ligation of LPS to TLR4 receptors on BV-2 microglial cells, downstream signaling pathways leading to the production of inflammatory cytokines TNF-α (e.g. ERK1/2 and NF-κB) and IL-6 (e.g. JNK, p38, and c-Jun) were different from each other and a specific compound might only modify one pathway without any effects on the other one (26, 30). Accordingly, it seems that in our study crocin was just able to modify the signaling pathway responsible for the regulation of TNF-α expression, but not IL-6. Not only in concurrent treatment with LPS+IFN-γ but also in all other treatment conditions examined in this study crocin could not modify IL-6 secretion in J774A1 macrophages regardless of their primary commitment state.

In our study, when cells were pretreated with crocin and then exposed to LPS/IFN-γ, *Nos2* mRNA and TNF-α secretion increased and *Arg1* mRNA decreased compared to untreated cells, and therefore, crocin could not prevent M1 induction by LPS+IFN-γ. Accordingly, when Zhang *et al*. pre-treated monocytes with the antioxidant butylated hydroxyanisole (BHA) before LPS and IFN-γ exposure, BHA could not inhibit the induction of the M1 state (31).

In untreated macrophages, crocin treatment did not cause any significant changes in the level* of Nos2* mRNA, while it decreased *Arg1* mRNA expression and increased TNF-α secretion, which was indicative of induction of an M1-like phenotype. In an *in vivo* study where crocin (40 mg/kg) was examined in mice to evaluate its protective effects against d-galactose-mediated oxidative stress and inflammatory response in healthy control animals, it significantly increased the TNF-α level in serum, but not the IL-6 level (32). In an earlier study designed to evaluate the protective effects of crocin against rat hepatic damage induced by D-galactose, crocin by itself (30 mg/kg/day, intraperitoneally) did not change *Nos2* protein levels in hepatic tissue of healthy control rats. However, when administered concurrently with D-galactose, crocin was able to inhibit D-galactose-mediated *Nos2* induction (33). Shen *et al*. demonstrated that crocin can decrease the differentiation of RAW264.7 cells into M2-like macrophages, and increase secretion of the cytokines associated with an M1-like phenotype (34). The same results have been reported for some other well-known antioxidants. The antioxidant Berberine isolated from *Coptis chinensis *suppressed intestinal tumorigenesis in the mouse model. This antitumor effect of berberine was shown to be mediated by a decrease in M2 macrophage polarization (35). Accumulating data revealed that ginsenosides as an antioxidant and the main active constituent of ginseng had the potential to efficiently alter TAM to the M1 subset of macrophages (36, 37). Since crocin has been used as a supplement in several clinical trial studies (38-42), these findings highlight that crocin is possibly able to modify the immune system of the recipient even in the absence of any inflammatory condition and also emphasize reassessing crocin administration as a preventive approach toward the diseases with a well-known etiology of M1 macrophage dominance.

It is noteworthy that we also tried to measure the secretion of interleukin 10 as a common marker for the M2 phenotype (43). However, it was undetectable in the supernatant of the cells in all experimental groups. Therefore, treatments of the J774A.1 murine macrophages with crocin and/or LPS+INF-γ could not induce any detectable levels of IL-10.

## Conclusion

It seems that crocin modulatory effects on macrophage M1/M2 phenotype switching are at least partly dependent on the primary commitment state of the cells when exposed to crocin. Crocin in concurrent treatment with M1-inducing mediators, LPS/IFN-γ, can suppress M1 induction. However, when crocin is solely applied to the macrophages, it acts as an M1 inducer by itself. Pretreatment of cells by crocin before the addition of LPS/IFN-γ, cannot converse the M1 induction in macrophages. Since crocin is widely used as an active component of some supplements in different countries, more detailed studies, especially in vivo investigations are required to clarify the differential immunomodulatory capabilities of crocin and the mechanisms involved in clinical health and disease states.

## Authors’ Contributions

H H conceived the original idea, secured funding, and approved the final version of the manuscript. F M designed the research proposal, interpreted data, participated in revising the article, and supervised the project. H A carried out the experiments, was involved in the analysis and interpretation of results, and wrote the manuscript with support from F M. M R and F M conducted the experiments and participated in analyzing and interpreting the outcomes.

## Conflicts of Interest

The authors declare that there are no conflicts of interest. 
